# Isolation and characterization of extracellular vesicles from biotechnologically important fungus *Aureobasidium pullulans*

**DOI:** 10.1186/s40694-022-00146-7

**Published:** 2022-11-01

**Authors:** Anja Černoša, Cene Gostinčar, Teja Lavrin, Rok Kostanjšek, Metka Lenassi, Nina Gunde-Cimerman

**Affiliations:** 1 Department of Biology, Biotechnical Faculty, University of Ljubljana, Jamnikarjeva 101, 1000 Ljubljana, Slovenia; 2 Institute of Biochemistry and Molecular Genetics, Faculty of Medicine, University of Ljubljana, Ljubljana, Slovenia

**Keywords:** Extracellular vesicles, *Aureobasidium pullulans*, Biocontrol, Proteomics, sRNA

## Abstract

Extracellular vesicles (EVs) are increasingly recognized as an important mechanism for cell–cell interactions. Their role in fungi is still poorly understood and they have been isolated from only a handful of species. Here, we isolated and characterized EVs from *Aureobasidium pullulans*, a biotechnologically important black yeast-like fungus that is increasingly used for biocontrol of phytopathogenic fungi and bacteria. After optimization of the isolation protocol, characterization of EVs from *A. pullulans* by transmission electron microscopy (TEM) revealed a typical cup-shaped morphology and different subpopulations of EVs. These results were confirmed by nanoparticle tracking analysis (NTA), which revealed that *A. pullulans* produced 6.1 × 10^8^ nanoparticles per milliliter of culture medium. Proteomic analysis of EVs detected 642 proteins. A small fraction of them had signal peptides for secretion and transmembrane domains. Proteins characteristic of different synthesis pathways were found, suggesting that EVs are synthesized by multiple pathways in *A. pullulans*. Enrichment analysis using Gene Ontology showed that most of the proteins found in the EVs were associated with primary metabolism. When sequencing the small RNA fraction of *A. pullulans* EVs, we found two hypothetical novel mil-RNAs. Finally, we tested the biocontrol potential of EVs from *A. pullulans*. The EVs did not inhibit the germination of spores of three important phytopathogenic fungi—*Botrytis cinerea*, *Colletotrichum acutatum*, and *Penicillium expansum*. However, exposure of grown cultures of *C. acutatum* and *P. expansum* to *A. pullulans* EVs resulted in visible changes in morphology of colonies. These preliminary results suggest that EVs may be part of the antagonistic activity of *A. pullulans*, which is so far only partially understood. Thus, the first isolation and characterization of EVs from *A. pullulans* provides a starting point for further studies of EVs in the biotechnologically important traits of the biocontrol black fungus *A. pullulans* in particular and in the biological role of fungal EVs in general.

**Supplementary Information:**

The online version contains supplementary material available at 10.1186/s40694-022-00146-7.

## Introduction

*Aureobasidium pullulans* (de Bary) G. Arnaud is a polyextremotolerant black yeast-like fungus known mainly for its biotechnological importance [1, 2]. As a ubiquitous fungus, *A. pullulans* is found in a wide range of environments, from polar to tropical, and both indoors and outdoors [1]. This can be attributed to the ability of *A. pullulans* to survive various growth-limiting conditions, such as acidic and alkaline [3], low temperature [4], hypersaline [5], and oligotrophic conditions [6].

In biotechnology, *A. pullulans* is important because of the production of the extracellular polysaccharide pullulan, which has applications in many different fields, for example in pharmacy for capsules and in the food industry as a stabilizer [7]. *A. pullulans* is also known as a producer of various extracellular enzymes (amylases, cellulases, lipases, proteases, etc.) that are biotechnologically useful [2].

In the search for more environmentally friendly solutions in agriculture, the antagonistic ability of *A. pullulans* is coming to the fore. The use of biocontrol agents is an important substitute for synthetic fungicides, which can have harmful effects on human health and the environment [8] and can lead to cross-resistance with medical antifungals [9]. There are numerous studies on the antagonistic activity of *A. pullulans* against various phytopathogenic fungi, e.g., *Penicillium expansum*, *Botrytis cinerea*, *Colletotrichum acutatum*, *Monilinia fructicola*, *Alternaria alternata*, *Monilinia* spp. on various fruits and vegetables, e.g., apples, grapes, strawberries, kiwifruit, stone fruit, tomatoes [10–20]. Several freeze-dried strains of *A. pullulans* are commercially available for use in biocontrol, e.g., AureoGold^®^, Blossom Protect^®^, and Boni Protect^®^ [21].

What makes *A. pullulans* a successful biocontrol agent is its great ability to displace other microorganisms by outcompeting them for nutrients and space [22, 23]. For example, *A. pullulans* can produce siderophores that can inhibit the growth of other species [21, 24–26]. It also synthesizes various extracellular enzymes that can act on the fungal cell wall, including chitinases and glucanases, and proteases that affect spore germination [10, 21, 27–29]. Other strategies that also play a role in the antagonistic activity of *A. pullulans* include the synthesis of various secondary metabolites (e.g., the antibiotic aureobasidin A) [30] and biofilm production [24, 31]. There is also evidence that *A. pullulans* can induce resistance in plants against the phytopathogen [32]. Many of these strategies involve excretion of active compounds into the environment and communication between the biocontrol agent, the phytopathogenic fungus, and the plant. The mechanism of this communication is largely unknown, but might involve extracellular vesicles.

Extracellular vesicles (EVs) are membranous vesicles that are ubiquitously secreted into the extracellular space by cells from all kingdoms. Most studies have focused on mammalian EVs [33]. The isolation and characterization of fungal EVs has long been neglected [34], in large part due to difficulties in isolation methodology. The first isolation and characterization of fungal EVs dates back to 2007, when EVs from the fungal pathogen *Cryptococcus neoformans* were described [35]. To date, fungal EVs have been isolated and characterized from only 25 different fungal species, most of which are medically important and only a few of biotechnological relevance (reviewed in [34]).

The main purpose of EV production and release is communication and signaling between cells from the same or different kingdoms [36]. By transmitting information from one cell to another, EVs influence neighboring cells [37]. EVs can contain various molecules, from proteins and nucleic acids to lipids, glycans, polysaccharides, and pigments [35, 38–43]. Their different molecular compositions also enable different functions (reviewed in [34]).

The best studied example of the interaction between phytopathogenic fungal EVs and plants is the role of EVs in virulence. For example, EVs from the cotton pathogen *Fusarium oxysporum* and the citrus pathogen *Penicillium digitatum* have phytotoxic effects on infected plants [43, 44]. Proteomic analysis of EVs from *Fusarium graminearum*, *Phytophthora capsica*, and *Zymoseptoria tritici* revealed proteins associated with plant pathogenicity [45–47]. Transcriptome analysis of EVs from *Ustilago maydis* also identified sequences encoding effectors associated with virulence [48]. On the other hand, plant-derived EVs can reduce infections with phytopathogenic fungi [49], mostly with different small RNA (sRNA) that can silence fungal genes associated with virulence [50]. In the case of *Phytophthora* infection of *Arabidopsis*, there appears to be a dynamic exchange of different EVs cargo between the plant and the fungi [51]. While these examples demonstrate that EVs can play an important role in the interactions between phytopathogens and plants, the role of the third partner in this relationship remains unknown: to date, no EVs have been isolated from biocontrol fungi and tested for their biocontrol potential.

In this article, we present the isolation protocol for EVs produced by *A. pullulans* and characterize EVs from this fungus for the first time. We also describe the proteins and sRNA found in EVs from *A. pullulans*. Finally, we present preliminary data on the potential biocontrol properties of EVs from *A. pullulans* on the phytopathogenic fungi *P. expansum*, *B. cinerea*, and *C. acutatum*.

## Materials and methods

### Strains and growth conditions

The type strain *Aureobasidium pullulans* (EXF-150) and the phytopathogenic fungi *Botrytis cinerea* (EXF-656), *Colletotrichum acutatum* (EXF-11123), and *Penicillium expansum* (EXF-12111) were obtained from the Culture Collection Ex of the Infrastructural Centre Mycosmo (Department of Biology, Biotechnical Faculty, University of Ljubljana, Slovenia) (Table 1). Cultures were maintained on malt extract agar (MEA) consisting of 2% malt extract (Biolife, Italy), 0.1% peptone (Merck, Germany), 2% glucose (Kemika, Croatia), and 2% agar (Formedium, UK) in deionized water.

**Table 1:** List of strains used in this study

Species	Culture collection strain number	Isolation habitat	Geographic location
*Aureobasidium pullulans*	EXF-150	Hypersaline water from saltpans	Slovenia, Portorož
*Botrytis cinerea*	EXF-656	Chardonnay grapes	Slovenia; Drašiči
*Colletotrichum acutatum*	EXF-11123	Rotten apple	Slovenia; Ljubljana
*Penicillium expansum*	EXF-11121	Rotten apple	Slovenia; Ljubljana

### Isolation of extracellular vesicles (EVs)

Isolation of EVs was performed as described in [35] with some modifications.

One loop of *Aureobasidium pullulans* culture was inoculated into 20 mL of liquid yeast nitrogen base (YNB) medium (pH 7.0) consisting of 0.17% yeast nitrogen base (Qbiogene, USA), 0.5% ammonium sulphate (SigmaAldrich, USA) and 2% glucose (Fisher Scientific, USA) in deionized water. The culture was incubated at 24 °C on a rotary shaker (180 rpm). After one day of incubation, the entire culture was inoculated into 100 mL of liquid YNB medium and incubated at 24 °C on a rotary shaker (180 rpm) until the middle of the exponential growth phase (OD_600_ = 0.8–1.5) was reached. Cells were then harvested by centrifugation at 1000×*g* for 10 min at 24 °C, washed three times in fresh YNB medium, and adjusted to OD_600_ = 0.2 in 400 mL YNB medium. After 24 h of incubation at 24 °C on a rotary shaker (180 rpm), when the culture reached the stationary growth phase (OD_600_ = 2.0–3.0), EVs were collected from the medium. Cells were removed by centrifugation at 4000×*g* for 15 min at 4 °C. The supernatant was filtered through a 0.22 µm PES filter (TPP, Switzerland) to remove polysaccharides and other larger particles. The filtrate was concentrated tenfold in the Amicon Stirred Cell Concentrator (Merck Millipore, Germany) using a 300 kDa PES membrane. The EVs were then pelleted by ultracentrifugation at 100,000×*g* for 1 h 10 min at 4 °C (Type 50.2 Ti, Optima XPN-80, Beckman Coulter, USA). The pelleted EVs were washed with DPBS (Dulbecco’s phosphate buffered saline; Sigma Aldrich, USA) and ultracentrifuged again under the same conditions (TLA-55, Optima MAX-XP, Beckman Coulter, USA). The final pellet was resuspended in 100 µL DPBS, aliquoted, frozen in liquid nitrogen, and stored at − 80 °C until further use. An exception was the aliquots for the functional tests, which were stored at 4 °C and used the same day.

Alternatively, EVs pelleted by the first ultracentrifugation step were suspended in 50 µL DPBS and frozen at − 20 °C until the next day, when the samples were thawed on ice and we added 350 µL DPBS. Then we centrifuged the samples at 100,000×*g* for 18 h at 4 °C (MLS-50, Optima MAX-XP, Beckman Coulter, USA) on a sucrose density gradient consisting of 400 µL fractions of 20%, 24%, 28%, 32%, 36%, 40%, 44%, 48%, 52%, 56%, and 60% sucrose (w/v), (Sigma Aldrich, USA) in DPBS. After ultracentrifugation, each fraction (400 µL) was collected, frozen in liquid nitrogen and stored at − 80 °C until further use.

### Characterization of extracellular vesicles (EVs)

#### Transmission electron microscopy (TEM)

EV-enriched samples were visualized by TEM using the negative staining method. The sample was applied to Formvar-coated and carbon-stabilized copper grids and contrasted with a 1% (w/v) water solution of uranyl acetate. Samples were examined using a CM100 (Philips, Amsterdam, The Netherlands) transmission electron microscope, operating at 80 kV. Images were recorded with Orius 200 camera (Gatan) and processed by DigitalMicrograph software version 2.32 (Gatan Inc., Pleasanton, CA, USA).

#### Nanoparticle tracking analysis (NTA)

The concentration and size of nanoparticles in EV-enriched samples were determined by nanoparticle tracking analysis (NTA) using NanoSight NS300 instrument (with 488 nm laser) connected to an automated sample assistant (both Malvern Panalytical). Samples from three biological replicates were diluted 10,000 times in PBS and imaged five times with camera level 15. The images were then visually inspected and excluded from analysis if major errors were detected. The raw data were analyzed using the NanoSight NTA 3.3 program with the following settings: detection threshold 5, water viscosity, temperature 25 °C, automatic settings for minimum expected nanoparticle size and blur, and minimum track length 10. Output data were expressed as EV concentration, i.e., the number of nanoparticles per 1 mL of medium, and EV size, i.e., the mean, modal, and median hydrodynamic diameter in nm.

Statistical analysis was performed using GraphPad Prism 9.3.1 software (GraphPad Software, Inc, California, USA). All data are given as mean ± standard error (SE).

#### Quantification of melanin

Melanin content was determined by measuring absorbance at 400 nm (A_400_) in 96-well plates using Synergy2 reader (BioTek, Winooski, VT, USA) [52].

#### Protein extraction and quantification

Proteins in EV fractions from sucrose density gradient were diluted to 500 µL in distilled water and then extracted by trichloroacetic acid—sodium deoxycholate precipitation (TCA-DOC) with the addition of 50 µL each of 70% (w/v) TCA and 0.15% (w/v) DOC followed by vortexing. Samples were incubated for 10 min and then centrifuged at 13,000×*g* for 10 min at room temperature. The pellet was then washed with ice-cold acetone to remove excess TCA and resuspended in 30 µL of radioimmunoprecipitation assay (RIPA) buffer consisting of 0.1% (w/v) sodium dodecyl sulfate (SDS; Sigma Aldrich, USA), 0.5% (w/v) DOC (Sigma Aldrich), 1.0% (v/v) NP-40 (IGEPAL CA-630; Sigma Aldrich), and 0.1% (v/v) protease inhibitor (Thermo Fisher Scientific, USA) in DPBS. The total amount of proteins (µg) in EV-enriched samples isolated from 400 mL of culture was determined using the Pierce BCA Protein Assay Kit (Thermo Fisher Scientific, USA) according to the manufacturer’s instructions.

#### Mass spectrometry

For mass spectrometry, we lysed the EV-enriched samples after ultracentrifugation (three independent biological replicates) according to [53] with some modifications. We added 25 µL RIPA buffer (Thermo Fisher Scientific, USA) containing 0.1% protease inhibitor (I3911-1BO, Sigma Aldrich, USA) to 1 µL of EVs samples and incubated the samples on ice for four hours. Then we carefully resuspended the sample for 15 min. After this, the protein concentration was determined using the Pierce BCA Protein Assay Kit (Thermo Fisher Scientific, USA) according to the manufacturer’s instructions.

The filter-aided sample preparation (FASP) protocol was performed using the FASP Protein Digestion Kit (Abcam, United Kingdom) according to the manufacturer’s instructions with some modifications. Briefly, 30 µL of the sample and 200 µL of the Urea Sample Solution were pipetted onto the Spin Filter and then centrifuged at 14,000×*g* for 15 min at room temperature. Then, another 200 µL of the Urea Sample Solution was pipetted onto the Spin Filter and centrifuged again under the same conditions. We then added 10 µL of the 10 × Iodoacetamide Solution and 90 µL of the Urea Sample Solution to the Spin Filter and vortexed the samples for one minute. The samples were then incubated in the dark for 20 min. After incubation, we centrifuged the samples at 14,000×*g* for 10 min at room temperature. We then pipetted 100 µL of the Urea Sample Solution onto the Spin Filter and centrifuged again at 14,000×*g* for 15 min at room temperature. We repeated this step one more time. Then, we added 100 µL of the 50 mM Ammonium Bicarbonate Solution to the Spin Filter and centrifuged the samples at 14,000×*g* for 10 min at room temperature. This step was repeated one more time. Then, 75 µL Digestion Solution (concentration of trypsin 0.25 µg/µL; Merck, Germany) was dropped onto the Spin Filter and the sample was vortexed for one minute. We incubated the sample at 37 °C for 18 h. The next day, we added 40 µL of 50 mM Ammonium Bicarbonate Solution to the Spin Filter and centrifuged at 14,000×*g* for 15 min at room temperature. This step was repeated one more time. We then transferred the Spin Filter to a new collection tube and added 50 µL of 0.1% formic acid in HPLC grade water to the Spin Filter. The samples were centrifuged at 14,000×*g* for 10 min at room temperature. The supernatant contained digested proteins. The supernatant was acidified with 2 µL of 0.5% TFA and then stored at − 20 °C until use.

The LC–MS/MS analysis was performed at Advanced Mass Spectrometry Facility in the School of Biosciences at the University of Birmingham. UltiMate^®^ 3000 HPLC series (Dionex, Sunnyvale, CA USA) was used for peptide concentration and separation. Samples were trapped on precolumn, Acclaim PepMap 100 C18, 5 µm, 100 Å 300 µm i.d. × 5 mm (Dionex, Sunnyvale, CA USA) and separated in Nano Series™ Standard Columns 75 µm i.d. × 15 cm, packed with C18 PepMap100, 3 µm, 100 Å (Dionex, Sunnyvale, CA USA). The gradient used was from 3.2% to 44% solvent B (0.1% formic acid in acetonitrile) for 30 min. The column was then washed with 90% mobile phase B before re-equilibrating at 3.2% mobile phase B. Peptides were eluted directly (~ 350 nL min^−1^) via a Triversa Nanomate nanospray source (Advion Biosciences, NY, USA) into a QExactive HF Orbitrap mass spectrometer (ThermoFisher Scientific). The spray voltage of QE HF was set to 1.7 kV through Triversa NanoMate and heated capillary at 275 °C. The mass spectrometer performed a full FT-MS scan (m/z 380−1600) and subsequent HCD MS/MS scans of the 20 most abundant ions with dynamic exclusion setting 15S. Full scan mass spectra were recorded at a resolution of 120,000 at m/z 200 and ACG target of 3 × 10^6^. Precursor ions were fragmented in HCD MS/MS with resolution set up at 15,000 and a normalized collision energy of 28. ACG target for HCD MS/MS was 1 × 10^5^. The width of the precursor isolation window was 1.2 m/z and only multiply-charged precursor ions were selected for MS/MS. Spectra were acquired for 56 min.

The MS and MS/MS scans were searched against Uniprot database using Protein Discovery 2.2 software, Sequest HT algorithm (Thermo Fisher). Variable modifications were deamidation (N and Q), oxidation (M) and phosphorylation (S, T and Y). The precursor mass tolerance was 10 ppm and the MS/MS mass tolerance was 0.02 Da. Two missed cleavage was allowed, and data were filtered with a false discovery rate (FDR) of 0.01. Protein with at least two high confidence peptides were accepted as real hit.

Proteins with 2 or more unique peptides found in at least two biological replicates were used for analysis. The data is available at the PRIDE repository with the identifier PXD037103.

The proteins found in EV-enriched samples were matched to predicted proteins of *A. pullulans* EXF-150 (GenBank Accession Number AYEO00000000) with 'blastp' 2.9.0 + using the best match as identification. The proteins of *A. pullulans* EXF-150 were classified with PANTHER (Protein ANalysis THrough Evolutionary Relationships) Classification System using the PANTHER17.0 database and 'pantherScore' 2.2. Proteins identified in the EVs were checked for enrichment in PANTHER categories compared to the background of the whole *A. pullulans* proteome at http://www.pantherdb.org/. Statistical overrepresentation test was used to present the enrichment of proteins found in EVs, using the Fisher’s Exact test. The correction was done by calculation of False Discovery Rate. Charts of PANTHER categories were prepared in ‘ggplot2’ [54], part of R [55].

To identify proteins with signal peptides we used SignalP version 6.0 [56], and to define membrane-associated proteins we used TMHMM version 2.0 [57, 58].

#### Small RNA and bioinformatics analysis of micro-like RNAs

We prepared sRNA for RNA-seq from three independent biological replicates. First, RNA was isolated from EV-enriched samples using the RNeasy Mini Kit (Qiagen, Germany) and then treated with the RNeasy MinElute Cleanup Kit (Qiagen, Germany) according to the manufacturer's protocol to obtain fractions enriched in small RNA. We used the Agilent 4200 TapeStation System (Agilent Technologies, California, USA) to determine the quality and quantity of sRNA.

RNA-sequencing was performed by Novogene Co (Beijing, China). The cDNA libraries of the sRNAs were prepared and then sequenced using NovaSeq 6000 (Illumina, California, USA).

The raw reads were trimmed for adaptor sequences and quality trimmed (Phred Score > 25) with BBDuk, part of BBTools 38.96 (https://sourceforge.net/projects/bbmap/). Reads mapping to tRNA, rRNA, snRNA and snoRNA were removed with BBSplit following comparison with Rfam database version 14.7 [59]. Micro-like RNA (milRNA) sequences were identified with miRDeep2 version 2.0.1.3 [60] using default parameters. Targets of the identified milRNA sequences in *A. pullulans* were predicted with miRanda version 3.3. For the identification of conserved milRNA sequences among the sRNAs identified in EVs from *A. pullulans*, we used a database miRBase containing mature miRNA sequences from all organisms characterized so far [61]. The datasets generated and analyzed during the current study are available in the NCBI Sequence Read Archive (SRA) repository under BioProject accession number PRJNA892012.

### Biocontrol function of extracellular vesicles (EVs)

#### Effects of EVs on spores of phytopathogenic fungi

To test the biocontrol function of *A. pullulans* EVs on spores of phytopathogenic fungi, we first prepared the spore suspension of three phytopathogenic fungi (*Botrytis cinerea* (EXF-656), *Colletotrichum acutatum* (EXF-11123), and *Penicillium expansum* (EXF-11121); Table 1) in sterile DPBS. Spores were counted using the hemocytometer and adjusted to a concentration of 1.5 × 10^5^ spores/mL. We then added 20 µL of the EVs sample (7.2 × 10^8^ (SE ± 2.9 × 10^7^) nanoparticles per milliliter of culture medium) to 20 µL of the spore suspension. The same was done for the controls, using 20 µL of DPBS instead of the EVs sample. The samples were then mixed carefully to avoid damaging the EVs. The 10 µL of mixture was then spotted onto the center of the PDA plate. We inoculated three plates for each mixture. The plates were incubated at 24 °C for five days. After incubation, we measured the size of the colonies to determine the reduction in growth of the phytopathogenic strains.

#### Effects of EVs on phytopathogenic cultures

To test the biocontrol function of EVs isolated from *A. pullulans* on grown cultures, we inoculated the phytopathogenic strains *Botrytis cinerea (*EXF-656), *Colletotrichum acutatum* (EXF-11123), and *Penicillium expansum* (EXF-11121) in the center of PDA medium plates (Table 1). Cultures were incubated at 24 °C for one week so that growth on the plates was confluent. After incubation, we spotted 10 µL of a fresh sample with EVs (6.0 × 10^8^ (SE ± 1.6 × 10^7^) nanoparticles per milliliter of culture medium) on each culture in three replicates. We used sterile DPBS as a control. Plates were incubated at 24 °C for four days and then checked for changes.

## Results

### Characterization of extracellular vesicles

EVs were enriched from 400 mL of a stationary phase *A. pullulans* culture according to an established protocol [62], with membrane pore size optimized to accommodate higher concentrations of the extracellularly released polysaccharide pullulan.

**Fig. 1:**
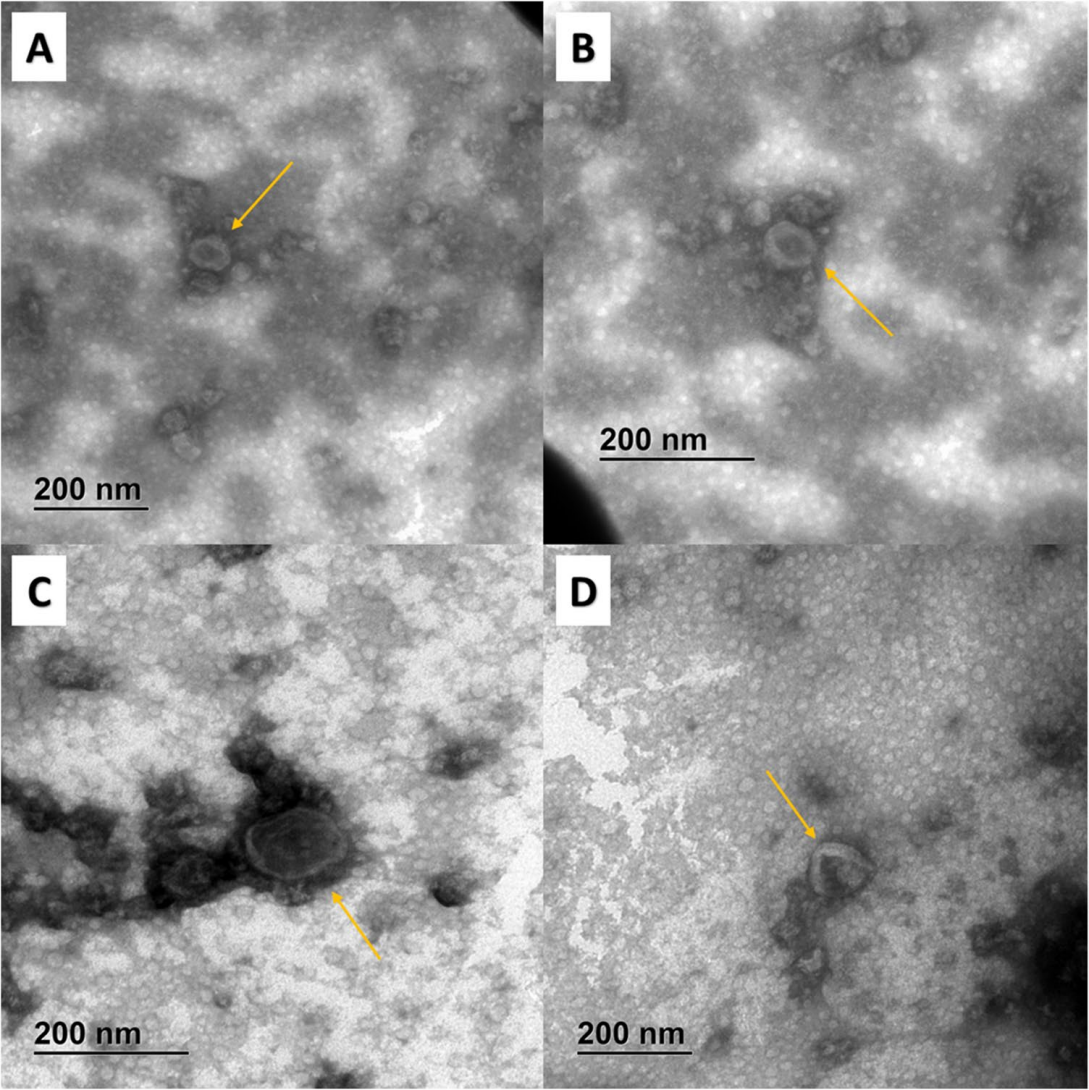
TEM micrographs of isolated EVs from *A. pullulans*. Images show different subgroups of EVs. The yellow arrows indicate the extracellular vesicles

**Fig. 2:**
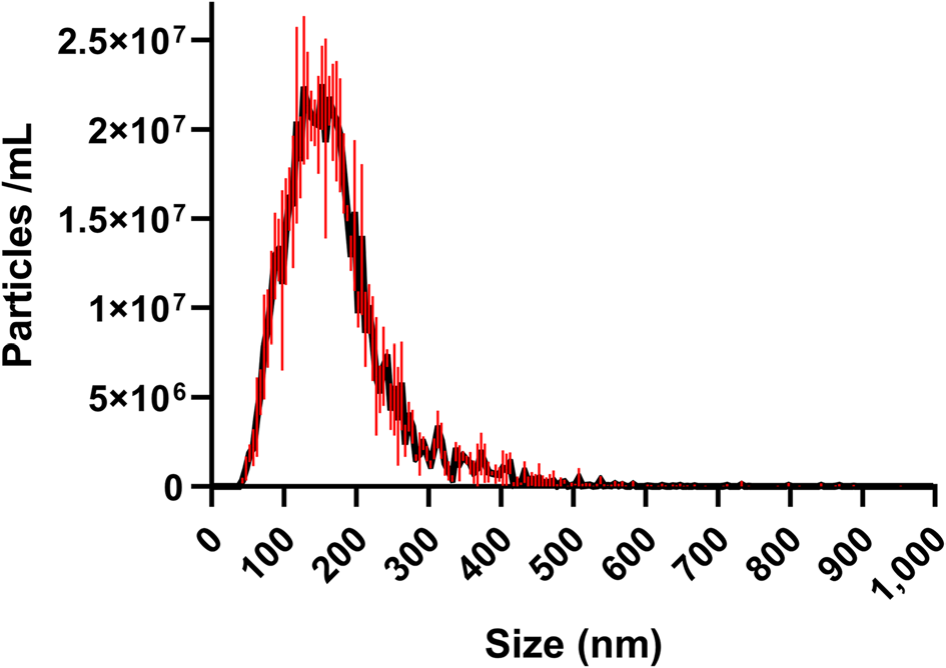
NTA detection of concentration and size distribution of *A. pullulans* EVs

Characterization of EVs isolated from *A. pullulans* was performed following MISEV2018 guidelines [63]. Transmission electron microscopy (TEM) revealed the typical cup-shaped morphology of dehydrated EVs (Fig. 1). Additional nanoparticles were isolated together with EVs. To quantify the nanoparticles in the EV-enriched samples, we performed nanoparticle tracking analysis (NTA). Most of the nanoparticles were in a size range between about 50 and 400 nm (Fig. 2). The fungus *A. pullulans* produced 6.1 × 10^8^ (SE ± 3.7 × 10^7^) nanoparticles per milliliter of 400 mL of culture medium. The average diameter of the nanoparticles was 171.4 nm (SE ± 3.7 nm).

**Fig. 3:**
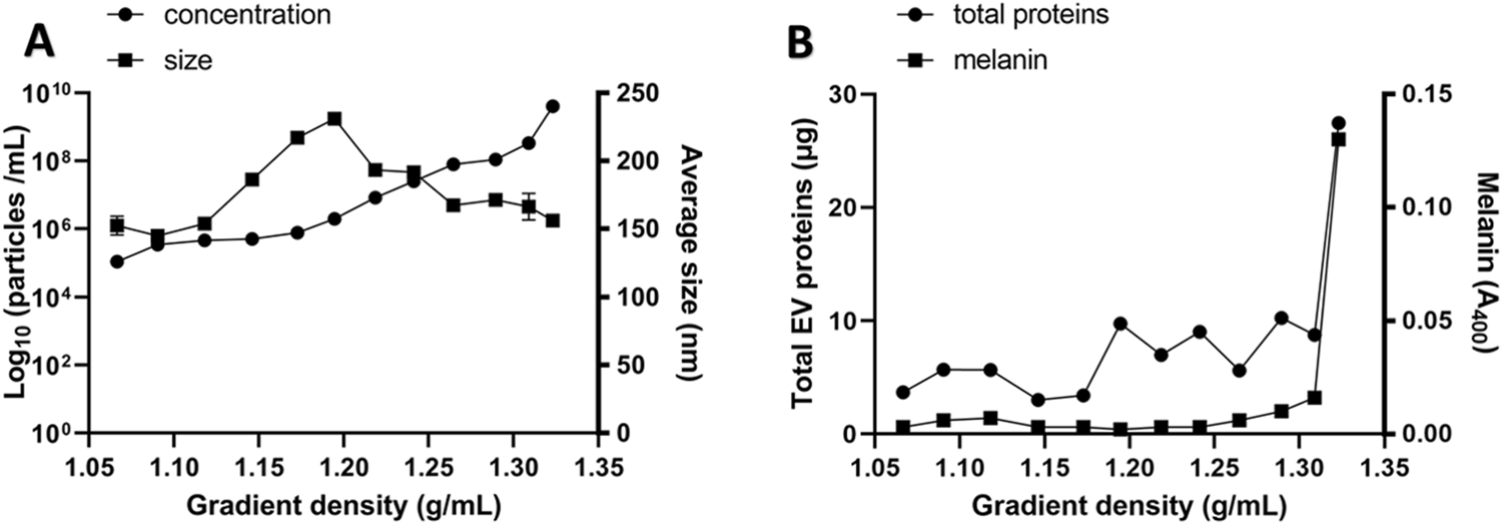
**A** NTA detection of concentration and size of nanoparticles and (**B**) distribution of total EV proteins (µg) and melanin content (A_400_) in different EV fractions isolated from *A. pullulans* by sucrose density gradient ultracentrifugation

In addition, we separated different subgroups of nanoparticles in the EV-enriched samples based on buoyant density ultracentrifugation. The number of nanoparticles found per milliliter increased with fraction density from 1.1 × 10^5^ (SE ± 1.3 × 10^4^) in the rarest fraction (density of 1.07 g/mL) to 4.1 × 10^9^ (SE ± 9.5 × 10^7^) in the densest fraction (density of 1.32 g/mL) (Fig. 3A). The average size of nanoparticles was similar in all fractions, with the average smallest nanoparticles in the second fraction (density of 1.09 g/mL) and the average largest nanoparticles in the sixth fraction (density of 1.20 g/mL). Each fraction contained different amounts of proteins, with the highest amounts in the densest fraction (density of 1.32 g/mL) (Fig. 3B). Melanin was present in small amounts in all fractions, but the majority was contained in a subset of the three densest fractions (densities of 1.29–1.32 g/mL).

### Proteins

Mass spectrometry analysis identified a total of 2099 different proteins in at least one of the three biological replicates of EV-enriched samples from *A. pullulans*. We decided to further process only those proteins that were present with at least two copies per sample in at least two of the three biological replicates. The resulting list contained 642 proteins (Additional file 1). Of the 642 proteins, 33 (5.1%) had transmembrane domains and 64 (10%) of them had secretion signals.

The 30 most abundant proteins found in EVs from *A. pullulans* were involved in various biological processes. The most abundant protein found was 5-methyltetrahydropteroyltriglutamate-homocysteine methyltransferase (A0A4S9AIT8), which is part of the methionine biosynthesis process (Table 2). Nine of the 30 most abundant proteins are related to carbohydrate metabolism (A0A4S8TEK2, A0A4T0BKW4, A0A074XKR6, A0A4S8WPV8, A0A4S8TCQ2, A0A1A7MK09, A0A4S9IG62, A0A4V4LMM1, and A0A1A7MP02). Five proteins are involved in stress response (A0A074XWL5, A0A4S9T4Y7, A0A074XJB5, A0A074YII9, and A0A4S9ITR5). Other proteins are part of signaling processes (A0A4S8V4V2), transport (A0A4V4KL85, A0A4S8WUD5), lipid and protein metabolism (A0A4S9AIT8, A0A4S9MT33, A0A1A7MKW1, A0A4S9ER00, A0A074XC67), oxidation/reduction reactions (A0A074X7J3, A0A4S9SUA4, A0A4S9LDW5), and translation (A0A1A7MQA9, A0A1A7MJ01).

**Table 2:** The 30 most abundant proteins identified in EVs from *A. pullulans*

Uniprot ID	Protein	Associated GO terms		
		Molecular function	Biological Process	Cellular Component
A0A4S9AIT8	5-Methyltetrahydropteroyltriglutamate-homocysteine methyltransferase	5-Methyltetrahydropteroyltriglutamate-homocysteine S-methyltransferase activity; zinc ion binding	methionine biosynthetic process	N/D
A0A074X7J3	NADP-specific glutamate dehydrogenase 1-related	Oxidoreductase activity	glutamate metabolic process; alpha-amino acid biosynthetic process	Cytosol
A0A4S8TEK2	Enolase	Hydro-lyase activity	glycolytic process	catalytic complex; cytosol
A0A074XWL5	Putative mitochondrial Hsp70 chaperone	ATP hydrolysis activity; ATP binding; heat shock protein binding; unfolded protein binding; misfolded protein binding	chaperone cofactor-dependent protein refolding; cellular response to unfolded protein	membrane; mitochondrion
A0A4T0BKW4	Glucose-6-phosphate isomerase	Small molecule binding; carbohydrate binding; isomerase activity	hexose biosynthetic process; glucose 6-phosphate metabolic process; glycolytic process; glucose metabolic process	cytosol
A0A074XKR6	Glyceraldehyde 3-phosphate dehydrogenase	Oxidoreductase activity, acting on the aldehyde or oxo group of donors, NAD or NADP as acceptor; NADP binding; F: NAD binding	glucose metabolic process	N/D
A0A4S8WPV8	Phosphoglycerate kinase	ATP binding; kinase activity	hexose biosynthetic process; glycolytic process; glucose metabolic process	cytosol
A0A4S8TCQ2	Transketolase	Transferase activity	generation of precursor metabolites and energy; glucose 6-phosphate metabolic process; NADP metabolic process	cytosol
A0A4S9SUA4	D-3-phosphoglycerate dehydrogenase 1-related	Oxidoreductase activity, acting on the CH-OH group of donors, NAD or NADP as acceptor; NAD binding	N/D	N/D
A0A4S9IG62	Glucokinase-1-related	Carbohydrate kinase activity; phosphotransferase activity, alcohol group as acceptor	Glucose 6-phosphate metabolic process; cellular glucose homeostasis; glycolytic process	membrane; mitochondrion; cytosol
A0A4S8V4V2	Inorganic diphosphatase	Inorganic diphosphatase activity; magnesium ion binding	phosphate-containing compound metabolic process	cytoplasm
A0A4T0E7S4	Protein MGARP	N/D	N/D	N/D
A0A4S9T4Y7	60 kDA heat shock protein	N/D	Protein folding	N/D
A0A4V4KL85	ATP synthase subunit beta	N/D	mitochondrial transmembrane transport; oxidative phosphorylation; mitochondrial transport; ATP synthesis coupled proton transport	Mitochondrial proton-transporting ATP synthase complex
A0A4S9MT33	ATP-citrate synthase	Acyltransferase activity	acyl-CoA metabolic process; amide biosynthetic process; purine ribonucleotide biosynthetic process; fatty acid biosynthetic process	Cytosol
A0A4S9LDW5	Inosine-5-monophosphate dehydrogenase related	Oxidoreductase activity, acting on the CH–OH group of donors, NAD or NADP as acceptor	purine ribonucleotide biosynthetic process; purine ribonucleoside triphosphate biosynthetic process	N/D
A0A1A7MQA9	60S ribosomal protein L4	RNA binding; structural constituent of ribosome	N/D	Cytosolic large ribosomal subunit
A0A1A7MK09	Pyruvate kinase	Intramolecular transferase activity; hydro-lyase activity; oxidoreductase activity; phosphotransferase activity, alcohol group as acceptor; kinase activity	glycolytic process	Cytoplasm
A0A074XJB5	Heat shock protein 83	Unfolded protein binding	cellular response to heat; protein folding; protein stabilization	Perinuclear region of cytoplasm; cytosol; plasma membrane
A0A074YII9	Hsc70Cb, isoform G-related	ATP binding; ATP-dependent protein folding chaperone	N/D	N/D
A0A1A7MKW1	Serine hydroxymethyltransferase	Carboxylic acid binding; zinc ion binding; heterocyclic compound binding; organic cyclic compound binding; small molecule binding; transferase activity, transferring one-carbon groups	Carboxylic acid catabolic process; cellular amide metabolic process; alpha-amino acid biosynthetic process; organonitrogen compound catabolic process; folic acid-containing compound metabolic process	Cytoplasm
A0A4S9ER00	ATP-citrate synthase	Acyltransferase activity	acyl-CoA metabolic process; amide biosynthetic process; purine ribonucleotide biosynthetic process; fatty acid biosynthetic process	Cytosol
A0A4S8WUD5	ATP synthase subunit alpha	ATP binding; proton transmembrane transporter activity; cation channel activity; ligase activity	ATP synthesis coupled proton transport	N/D
A0A074ZIJ5	Negative regulator of sporulation Mds3-related	N/D	Cellular homeostasis; regulation of cellular process	Membrane; mitochondrion; cytosol
A0A4S9ITR5	Ribosome-associated molecular chaperone Ssb1-related	ATP hydrolysis activity; ATP binding; heat shock protein binding; unfolded protein binding; misfolded protein binding	Vesicle-mediated transport; chaperone cofactor-dependent protein refolding; cellular response to unfolded protein	Nucleus; cytosol; plasma membrane
A0A4S9IQS0	Adenine phosphoribosyltransferase	Cation binding; anion binding; pentosyltransferase activity; purine ribonucleotide binding	Purine ribonucleotide biosynthetic process; nucleobase metabolic process; AMP metabolic process; pigment biosynthetic process	Cytoplasm
A0A1A7MJ01	Eukaryotic translation elongation factor 2	Translation elongation factor activity; GTPase activity; ribosome binding	Translational elongation	Cytosol; ribonucleoprotein complex
A0A074XC67	Polyketide synthase 1	Acyltransferase activity, transferring groups other than amino-acyl groups	Secondary metabolite biosynthetic process; fatty acid biosynthetic process	N/D
A0A4V4LMM1	Aconitate hydratase	Hydro-lyase activity; iron-sulfur cluster binding	Tricarboxylic acid cycle	Cytosol
A0A1A7MP02	Phosphohexomutase family member	Intramolecular transferase activity	Carbohydrate metabolic process	Cytosol

*N/D* no data

**Fig. 4:**
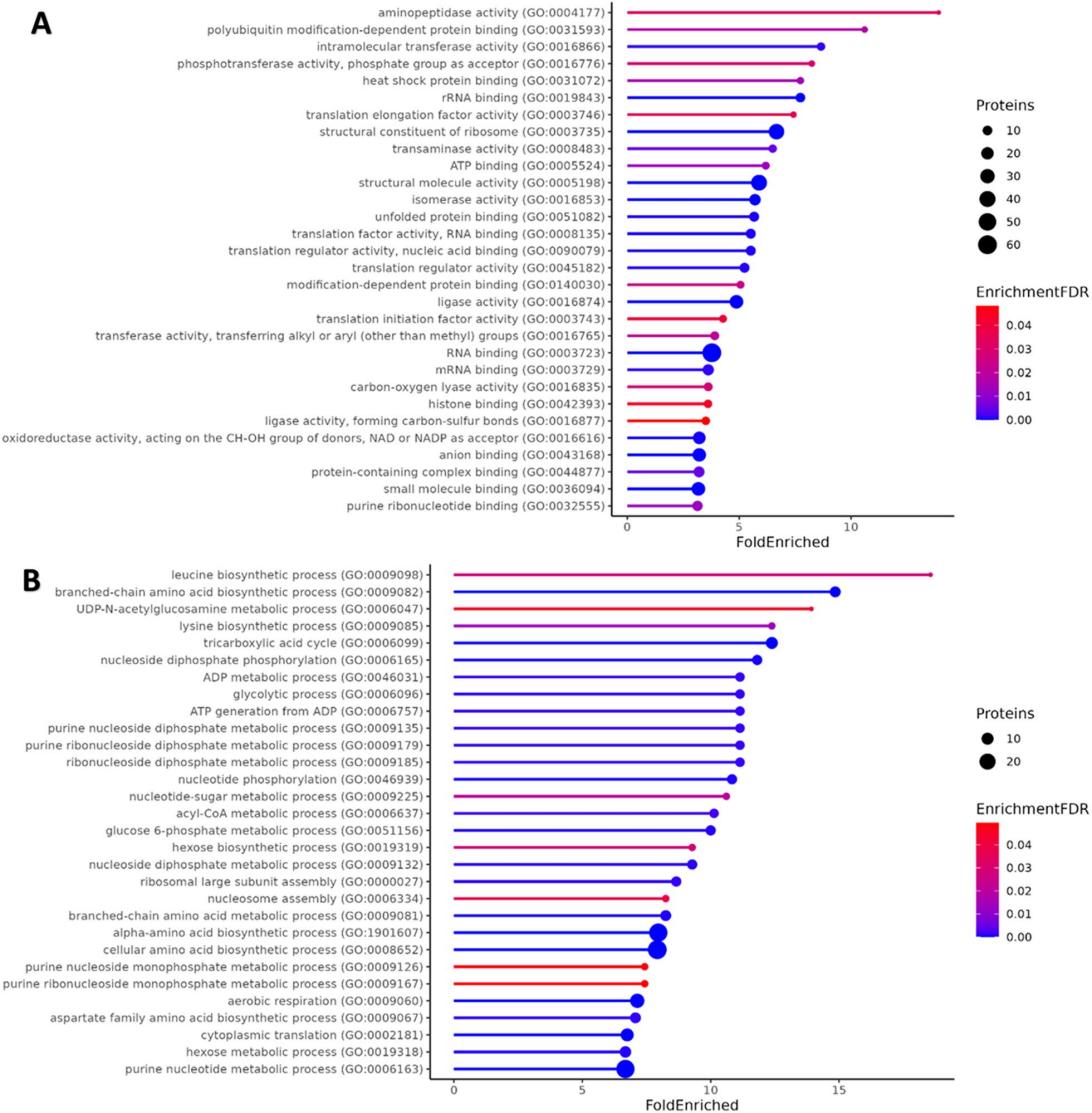
The top 30 GO terms overrepresented in *A. pullulans* EVs proteins in **A** Molecular Function, and **B** Biological Process. Encrichment FDR is the false discovery rate value of the enrichment analysis. The full list can be found in Additional file 2

Enrichment analysis of GO Molecular Functions showed that proteins related to primary metabolism, such as metabolism of proteins, hydrocarbons, and lipids, as well as proteins involved in translation and response to stress, were significantly overrepresented in EVs (Fig. 4). Underrepresented were functions related to transcription and transport (Additional file 2).

Analysis of GO Biological Processes showed that EVs were mainly enriched in proteins involved in biosynthesis of different amino acids, cell wall biogenesis, carbohydrate metabolism, and nucleotide metabolism (Fig. 4; Additional file 2).

### mil-RNA

**Fig. 5:**
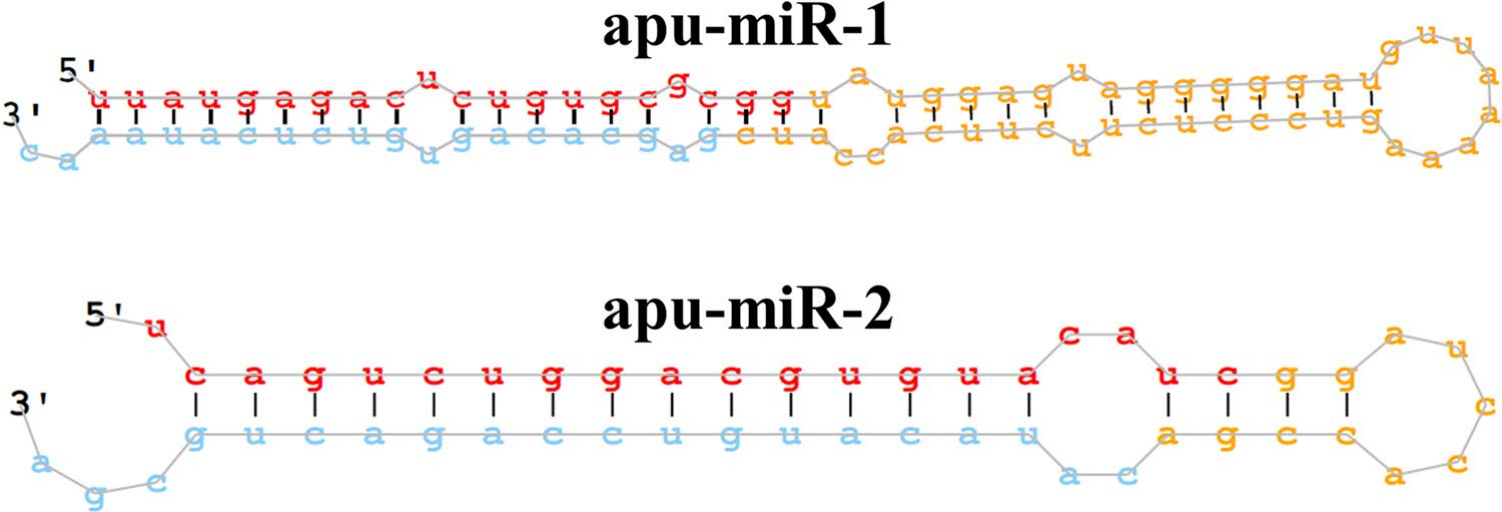
mil-RNA secondary structure predicted with miRDeep2 analysis. The sequence of the mature 5p miR is shown in red while the sequence of the mature 3p miR is shown in blue

The annotated *A. pullulans* genome contains annotations only for protein-coding regions [1], therefore miRNA-like RNAs (mil-RNA) were identified using miRDeep2. Our bioinformatic analysis of sequenced mil-RNAs predicted 33 unique mil-RNAs, but only two were found in all three replicates and in significant numbers. These two were assigned as apu-miR-1 and apu-miR-2 (Fig. 5; Additional file 3). We searched the miRBase database for the homologues of the two mil-RNAs but found no close homologues. The predicted targets for both mil-RNAs in *A. pullulans* were identified by miRanda. The targets of these two mil-RNAs are mainly hypothetical proteins with no known functions (Table 3).

**Table 3:** The 11 top targets for apu-miR-1 and apu-miR-2 in *A. pullulans* and their free energy (ΔG) based on miRanda

mil-RNA	Targets	ΔG
apu-miR-1	Thiamin diphosphate-binding protein	− 27.67
	Hypothetical protein	− 26.60
	APG17-domain-containing protein	− 27.38
	FAD/NAD(P)-binding domain-containing protein	− 27.39
	Hypothetical protein	− 25.07
	Aldo/keto reductase	− 18.91
	Hypothetical protein	− 19.92
	Putative liver carboxylesterase 4	− 41.21
	Hypothetical protein	− 22.64
	Hypothetical protein	− 44.08
	Hypothetical protein	− 29.65
apu-miR-2	Hypothetical protein	− 97.95
	NAD(P)-binding protein	− 32.53
	Hypothetical protein	− 28.32
	Hypothetical protein	− 30.52
	WSC-domain-containing protein	− 29.22
	Hypothetical protein	− 44.73
	Hypothetical protein	− 27.67
	Hypothetical protein	− 44.59
	Hypothetical protein	− 41.21
	Ribonuclease R	− 73.31
	Hypothetical protein	− 26.58

### Biocontrol function of extracellular vesicles

**Fig. 6:**
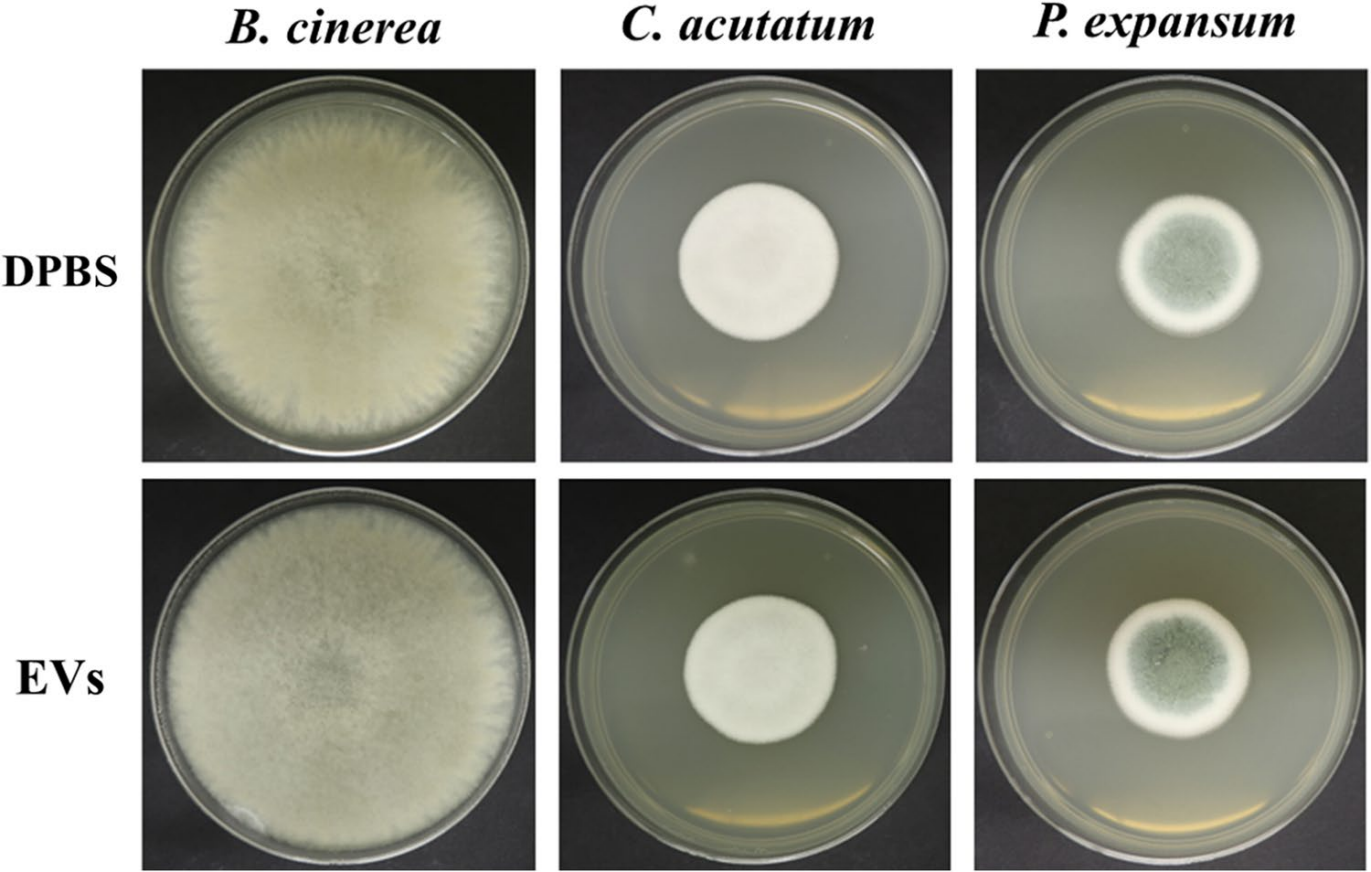
Results of testing the effect of *A. pullulans* EVs on spore germination of the phytopathogenic fungi *B. cinerea*, *C. acutatum*, and *P. expansum*. Fresh EVs sample (7.2 × 10^8^ (SE ± 2.9 × 10^7^) nanoparticles/mL of culture medium) was gently mixed with 1.5 × 10^5^ spores/mL in DPBS and then inoculated into the center of the PDA medium plate. Sterile DPBS was used as a control instead of the EVs sample. The plates were incubated at 24 °C for five days and then the size of the colonies was measured to determine whether the growth of phytopathogenic strains was reduced. The plate diameter was 90 mm

As a preliminary investigation into whether EVs produced by *A. pullulans* might play a role in the biocontrol potential of this species, we performed two different experiments. With the first experiment, we tested if the EVs from *A. pullulans* have an effect on the germination of spores of phytopathogenic fungi *B. cinerea*, *C. acutatum* and *P. expansum*. As demonstrated in Fig. 6, there was no reduction in spore germination for any of the tested phytopathogenic fungi after five days of incubation at 24 °C. All phytopathogenic fungi had the same colony diameter regardless of whether the spores were mixed with EVs or with sterile DPBS as a control prior to inoculation.

**Fig. 7:**
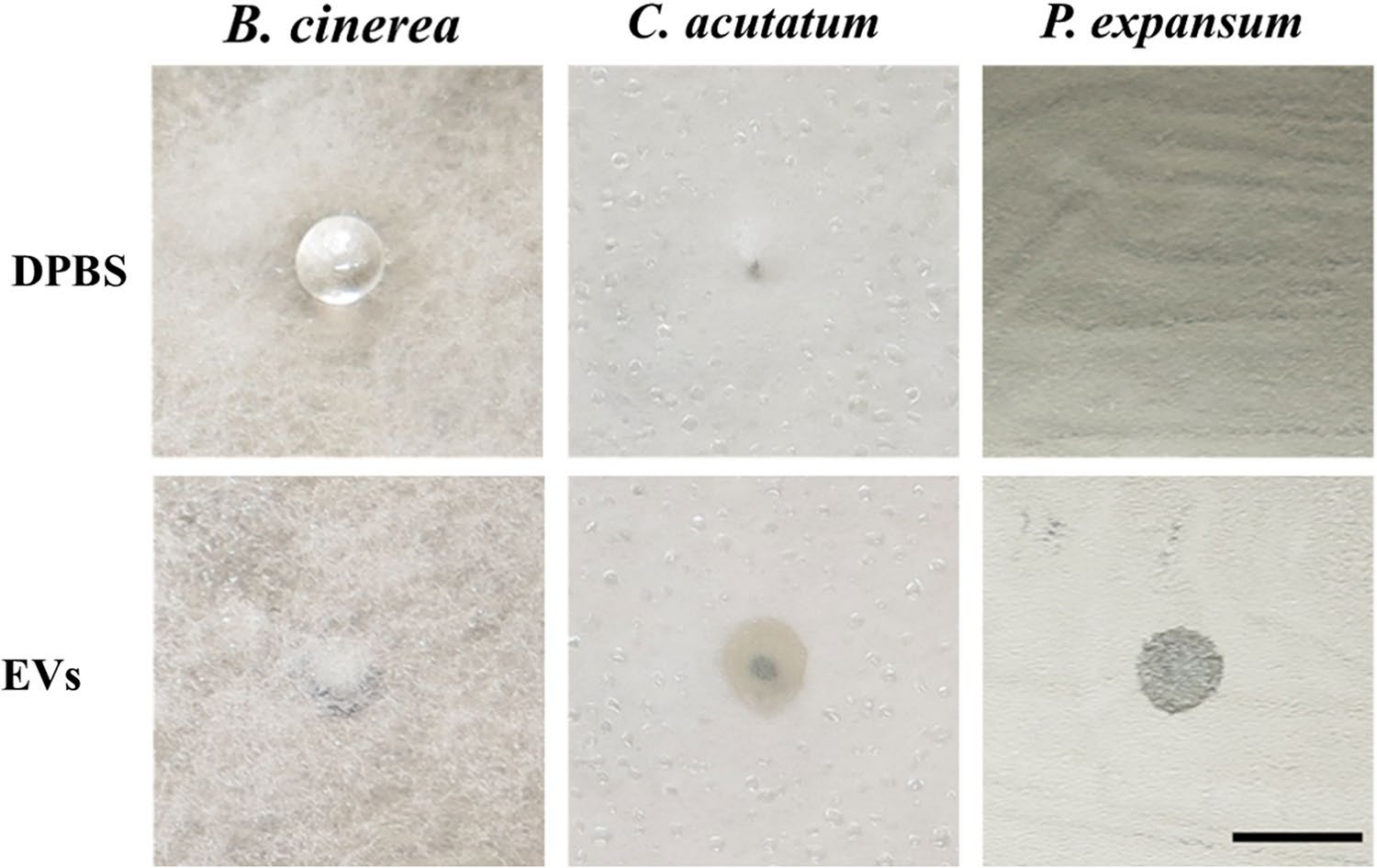
Test of the biocontrol potential of EVs from *A. pullulans* on three pathogenic fungi *B. cinerea*, *C. acutatum*, and *P. expansum*. Fungi were inoculated in the center of PDA medium plates and incubated at 24 °C until the growth on plates was confluent (one week). Then, a fresh EVs sample (6.0 × 10^8^ (SE ± 1.6 × 10^7^) nanoparticles/mL of culture medium) was spotten on cultures in three replicates. Sterile DPBS was also spotted on cultures in three replicates as a control. After four days of incubation at 24 °C, the cultures were examined for changes. Scale bar represents 0.5 cm

With the second experiment, we checked whether the EVs of *A. pullulans* have any effect on mature colonies of phytopathogenic fungi. EV-enriched samples were spotted onto cultures of phytopathogenic fungi and then examined for differences compared to the control (sterile DPBS). After incubation at 24 °C for four days, we found that there were some changes on the cultures of *C. acutatum* and *P. expansum* at the sites where the EV-enriched samples were spotted onto the cultures in comparison to the sites where we had spotted the controls (Fig. 7). It appears that the EV-enriched samples thinned out the cultures, as we see a black background, that we cannot observe on the control. We observed a drop of DPBS on the culture of *B. cinerea*, whereas there was no drop on the part of the culture to which the EVs sample was added. This phenomenon is probably due to the higher surface tension of DPBS. However, no difference was observed on the culture of *B. cinerea* between the EV-enriched samples and the control.

## Discussion

*Aureobasidium pullulans* is an extremotolerant, black, yeast-like fungus that plays an important role in biotechnology and is of increasing interest because of its use as a biocontrol agent in agriculture [1, 2, 7, 24]. In our study, we aimed to isolate and characterize EVs from *A. pullulans* (EXF-150) and provide tools for investigating the potential role of EVs in the substantial antagonistic activity of this species.

Since this was the first isolation of EVs from *A. pullulans*, we optimized the standard protocol for isolating fungal EVs from liquid cultures. Due to the high production of the extracellular polysaccharide pullulan, which increases the viscosity of the culture, we could not use the 100 kDa Amicon membrane normally used in the protocol [62]. Instead, we concentrated the sample with a 300 kDa Amicon membrane, assuming that pullulan would reduce the pore size of the membrane, thus retaining the EVs in the concentrated sample. Subsequent analyzes confirmed the viability of this approach.

After isolation of *A. pullulans* EVs, we followed the MISEV 2018 guidelines for characterization of EVs [63], although the guidelines for fungal EVs are relatively non-specific, as the basic understanding of fungal EVs is still lacking. Confirmation of successful isolation of EVs depended mainly on physical characterization, since there are no general biomarkers for fungal EVs. TEM confirmed the successful isolation of *A. pullulans* EVs, which exhibited a cup-shaped morphology, characteristic for dehydrated EVs and similar to previously isolated fungal EVs (Fig. 1) [44, 45, 47]. We also detected different subpopulations of extracellular nanoparticles in the same samples, which is also common when isolating EVs by ultracentrifugation. NTA confirmed the size diversity of EVs in our sample (Fig. 2). The concentration and average size of EVs isolated from *A. pullulans* apply to EVs in the size between 50 and 400 nm, because of the lower detection limit of NTA and because of the use of 0.22 µL membrane filter at the upper edge.The size of the nanoparticles detected was consistent with previous reports on the size of fungal EVs [44, 64, 65]. However, some studies show that fungal EVs can be larger than 400 nm [64, 66, 67]. The concentration of nanoparticles per milliliter of culture was similar to *Z. tritici*, but 10,000-fold lower than in *F. oxysporum* [44, 45].

EVs can contain different pigments, for example, EVs from *F. oxysporum* contain purple pigments [44]. EVs from the black fungus *Exophiala dermatitidis* contain the pigment melanin [62]. Since *A. pullulans* is also known to produce melanin, although its amount varies greatly depending on strain and growth conditions [1], we checked whether it was present extracellularly. Therefore, we measured melanin in each fraction of the sample after sucrose density gradient ultracentrifugation. The highest amount of melanin was found in the densest fractions, which is comparable to the results of EVs from *E. dermatitidis* [62]. Melanin can affect the pathogenesis of phytopathogenic fungi, however in the case of *A. pullulans*, which is nonpathogenic, the production of melanin may confer resistance to environmental stress [68, 69].

We performed proteomic analysis of EV-enriched samples from *A. pullulans* to characterize the proteins and see whether those proteins could be linked to biocontrol potential of the species. By analyzing three independent biological replicates, we aimed to reduce the impact of noise and obtain results with higher confidence, since omics data depend mainly on rigorous statistical analysis [70]. We identified 642 proteins present in at least two biological replicates, which is more than in EVs isolated from *Alternaria infectoria*, *C. neoformans*, *F. oxysporum*, *Z. tritici, P. capsica*, and *Saccharomyces cerevisiae* [38, 44–46, 71, 72]. As Bleackley and colleagues suggested, the higher number of proteins identified likely better reflects the reality of cargo in fungal EVs [70]. However, since we detected additional nanoparticles in our EV-enriched samples, some of the proteins could also be associated with those. Nevertheless, the percentage of proteins with signal peptides for secretion was similar to previous analyzes of signal peptides in fungal EVs [45, 70]. However, the number of proteins with transmembrane domains in EVs was lower than previously reported [45].

The 30 most abundant proteins mainly belong to categories of primary metabolism and stress response, which is a very common protein profile in fungal EVs [70]. However, from the perspective of biocontrol function, another interesting protein was found in *A. pullulans* EVs – the negative regulator of sporulation Mds3 (Table 2). The Mds3 protein has been generally studied in *S. cerevisiae*, where it negatively regulates early sporulation-specific genes for ascospores [73, 74].

Proteins belonging to different synthesis pathways of fungal EVs were present in lower numbers. Several proteins could be associated with pathways involved in the biogenesis of EVs: the ESCRT-III component, which is associated with the regulation of exosome synthesis (Additional file 1) [75]; proteins belonging to the coat protein complex (COP), which is part of the small vesicles that mediate the transport of proteins between different organelles; proteins associated with the Golgi synthesis pathway (Additional file 1) [75]. Taken together, these data suggest that EVs from *A. pullulans* are synthesized through several independent pathways.

Various researchers have previously attempted to identify common biomarkers for fungal EVs. Dawson and colleagues have suggested that the most appropriate biomarkers for EVs from *C. albicans* are the Sur7 and Evp1 proteins, claudin-like proteins from the Sur7 family, but neither of these proteins was detected in our proteomic analysis [64]. However, EVs from *A. pullulans* contained large amounts of different heat shock proteins, including Hsp70, which is normally found in mammalian EVs (Table 2; Additional file 1) [70]. Unfortunately, the Hsp70 protein cannot be used alone as a biomarker for fungal EVs because it can be present in multiple cellular locations and is not specific for EVs [70]. Since proteins in EVs differ by species, growth media, and other growth conditions, finding a general biomarker for all fungal EVs may prove to be extremely difficult, if not impossible.

Our enrichment analysis of proteins in *A. pullulans* EVs showed that most proteins are related to primary metabolism, which is common in fungal EVs (Fig. 4) [44, 45, 71, 72]. Proteins related to cell wall biogenesis, translation, and response to stress were also overrepresented in *A. pullulans* EVs, which is similar to findings in *F. oxysporum*, *P. capsici*, and *S. cerevisiae* [44, 46, 72].

Besides proteins, nucleic acids are also part of the cargo of EVs (reviewed in [76]). EVs can protect RNA from RNase degradation, allowing post-transcriptional regulation of neighboring cells at the level of gene expression [77]. The most commonly studied EV-associated RNAs in fungi are mRNA, microRNA-like (mil-RNA), and non-coding RNAs [77]. EVs-associated RNAs are also important for fungal phytopathogenesis [78]. Therefore, we wanted to investigate mil-RNAs in the EVs of *A. pullulans* and determine whether they could be important for the antagonistic activity of *A. pullulans* against phytopathogenic fungi. In our study, only two mil-RNAs were found in all three biological replicates (in abundance), which is less than in other fungal EVs [67, 77, 79] and suggests that the production and secretion of sRNA is not common under the growth conditions used. As shown by the research of Leone et al. [80], growth conditions affect the production and secretion of EVs containing sRNA [80]. Most of the predicted potential targets of the two mil-RNAs in *A. pullulans* were hypothetical proteins even though the genome is otherwise reasonably well-annotated. This makes it difficult to speculate about the role of the sRNAs found in the EVs from *A. pullulans*. Furthermore, since mil-RNAs may have targets in organisms other than *A. pullulans* itself (e.g., other fungi, plants), the identification of the EVs mil-RNAs represents just the first step in understanding micro-like RNAs in *A. pullulans* EVs.

The discovery that EVs from phytopathogenic fungi play a role in plant pathogenesis and that there is some cross-communication between plants and fungi during infection has led us to perform preliminary investigation into the possible effects of EVs on the growth of other fungal species. The culture from which the EVs were isolated was not stimulated by the presence of other fungi, but that is not always a requirement for the production of compounds involved in interspecies interactions. For example, some studies suggest that the virulence function of EVs of phytopathogenic fungi does not depend on the co-cultivation of phytopathogenic fungi and plants [44, 45, 47, 48]. Furthermore, there is some evidence that the cell filtrate of *A. pullulans* has biocontrol potential even when the species is cultivated independently of the presence of phytopathogens [81].

In our setting *A. pullulans* EVs did not inhibit the germination of spores of the selected phytopathogenic fungi (Fig. 6). However, when *A. pullulans* EVs were applied directly to growing colonies of phytopathogenic fungi, there were some indications of antagonistic activity against two of the three tested phytopathogens (Fig. 7). Further research is needed to confirm and characterize the potential biocontrol activity of *A. pullulans* EVs during different stages of phytopathogen growth and it should investigate various additional factors that could influence the cargo of EVs and have an impact on their biocontrol activity. These factors include the growth medium, temperature, lighting, growth time, and particularly cultivation in the presence of phytopathogenic fungi. The latter would require that EVs of *A. pullulans* can be distinguished from EVs of phytopathogenic fungi—a task that is currently difficult, if not impossible, to perform. Researchers attempting to isolate EVs from phytopathogenic fungi in an in vivo culture face the same problem [43]. In further studies it would be interesting to see whether different subpopulations of EVs isolated by density gradient ultracentrifugation or size-exclusion chromatography will generate different results.

In summary, we report for the first time the successful isolation and characterization of EVs from *A. pullulans*, a fungus of increasing importance in biotechnology and biocontrol. EVs produced by the fungus under standard growth conditions contain unusually large number of proteins and unusually few mil-RNAs compared to other fungi. Our results offer some indication that EVs may play a role in biocontrol potential of *A. pullulans* and provide a starting point for future analyzes of EVs as a mechanism of antagonism between species of the fungal kingdom.

## Supplementary Information

**Additional file 1**: List of all 642 proteins detected by mass spectrometry in EV-enriched samples from *A. pullulans*.

**Additional file 2:** The GO terms overrepresented and underrepresented in *A. pullulans* EVs proteins in Molecular Function, and Biological Process.

**Additional file 3:** The information of mil-RNAs apu-miR-1 and apu-miR-2 by miRDeep2.

## Data Availability

Data generated or analyzed during this study are included in this published article and its supplementary information files and are also available at the PRIDE repository (identifier PXD037103) an the NCBI SRA repository (BioProject accession number PRJNA892012).
